# Preservatives in Foods

**Published:** 1909-11-20

**Authors:** James Oliver

**Affiliations:** Physician to the Hospital for Women, London


### SPECIAL ARTICLE.

### PRESERVATIVES IN FOODS.

By JAMES OLIVER, M.D., F.R.S. (Edin.); Physician to the Hospital for Women, London.

The incorporation of chemical preservatives with foodstuffs is an ever-increasing evil, a nefarious and fraudulent practice requiring on the part of hygienists the greatest vigilance, and calling for the strictest action on the part of our Legislature. Public interest in this very important matter must not only be aroused, but energetically maintained; for quite needlessly, as well as unwittingly, we are all exposed and subjected to the pernicious influences of these toxic agents; insidiously and stealthily, derangement of every function of our bodies may thus be induced. Sufficient protection is not, as has been thoughtlessly alleged, afforded by our legislators fixing a maximal limit for one or more chemical preservatives in a given food; the danger lies pre-eminently in the fact that under such circumstances small quantities of these highly active substances may be ingested with a variety of foods which are in constant daily use, and it must not be forgotten that in our bodies the preservatives themselves or their effects are cumulative. Moreover, on account of idiosyncrasy some individuals are much more susceptible than others to the prejudicial influence of these agents.

From time immemorial the use of some preservatives in certain food products has been customary and permissible, but the most important of these—like salt, sugar, vinegar, and wood-smoke—are chiefly, if not entirely, condiments, and reveal their presence by taste or odour. The up-to-date preservatives are of a totally different category, for to be of any commercial value—and this is their prime function—they must elude detection by the consumer. Moreover, in consequence of the strict enforcement now, for their own domestic purposes, of the Foods and Drugs Act in the United States, the makers and users of modern preservatives consider it incumbent on them from personal motives to adopt preservatives which when incorporated with foods may tax the ingenuity of the analyst. It is a significant fact that already makers have placed on the market preservatives which, it is alleged, so combine with the foods for which they are specially recommended that the chemist cannot detect them. I would here remark, however, that this undesirable attainment has not yet been achieved.

Many who approve of and advocate the use of modern preservatives contend, and desire us to believe, that these are in no respect so very different from the ancient varieties; and in extenuation of the employment of the former they adduce the fact that the majority of them are merely very ordinary, medicinal agents, and that some even—like benzoic acid, which exists in cranberries—occur naturally in products used occasionally as foods. These are not, however, legitimate and incontrovertible excuses; for no one in a state of health is eager and anxious to ingest daily and for ever chemical substances which are neither foods nor condiments, materials which, as a matter of fact, are foreign to the foodstuffs with which for mercenary purposes they have been incorporated. We are desired, moreover, to believe that foods treated with such preservatives are presented to the consumer in a more wholesome state, in the best possible condition, whereas in many cases the true position of affairs is that by means of modern preservatives manufacturers and producers are enabled to arrest putrefaction and decay as well as to restore the colour to stale meats and to put on the market foods which are in reality unfit for use, and had they not been thus treated would have been condemned. Taking everything into consideration, we are warranted in arriving at the conclusion that chemical preservatives were introduced not because of any benefits they are likely to confer upon the consumer, but purely and simply because of their inestimable value to the large manufacturer and producer. To the latter they are a source of wealth, and cannot without a struggle be abandoned, for through their agency food products can be accumulated and stored more economically than is otherwise possible. Take, for example, tomatoes, which are used in the manufacture of a great variety of relishes, etc. This fruit is bought in immense quantities during a short period in summer; if the pulp which is to be stored and kept for an indefinite length of time be not treated with some preservative, like benzoic acid or salicylic acid, it must forthwith be sterilised by heat. The former is an easy and inexpensive treatment, and confers this advantage, that the fruit may for stated purposes be dealt with leisurely and according to requirements; consequently it is more acceptable and is more generally adopted than the more expensive and elaborate treatment of sterilisation by heat. Again, by the use of sulphurous acid and the sulphites decay may be concealed, and the colour may be so restored to stale meat that the latter may be made to appear quite fresh. The same is true with regard to the preparation of evaporated and desiccated fruits, but in this case the preservative not only conceals and arrests decay, but allows of more water being retained by the fruit, and the additional weight resulting therefrom is an undoubted advantage to the producer. When preservatives are incorporated with food the benefits to the consumer are inappreciable, whilst the advantages to the manufacturer are enormous.

The most important of the up-to-date preservatives are boric acid, salicylic acid, benzoic acid, the sodium compounds of these, sulphurous acid and the sulphites, formaldehyde, hydroxyl, and fluoride of ammonium. When there are present in the foods exposed to the action of such a preservative as sulphurous acid organic radicles like aldehydes and sugar, then compounds are formed which are more or less stable; but it is an indisputable fact that all the modern chemical preservatives are more or less toxic substances, and in an unchanged or changed form the duty of freeing the body of them falls entirely upon the kidneys, which under ordinary circumstances are already overburdened with work; and it is most reprehensible to add heedlessly and unnecessarily to their labours. Renal insufficiency and albuminuria may thus be induced.

Our life, it must be remembered, depends upon ferments, upon the action of extra-cellular as well as intra-cellular ferments, and every one of the chemical preservatives when ingested with food passes readily into the blood and tends to modify and alter the action of every ferment in our bodies so that metabolism is thereby more or less seriously deranged. In the gastro-intestinal tract they tend to augment or diminish the secretive, selective, and absorptive powers of the cells concerned in assimilation, and to provoke indigestion with all its attendant distresses. It is noteworthy that even when they stimulate and arouse to greater activity the digestive apparatus and improve the appetite, loss of body weight may nevertheless be observed.

In the blood they exert a prejudicial influence upon the white and red cells, and as our natural immunity to disease depends very especially upon the vigour and efficiency of the white blood corpuscles it is evident that the indiscriminate introduction into our bodies of chemicals which adversely affect the phagocytic power of the cells is a matter of the gravest import. Acting upon the tissues they hasten or retard the processes of oxidation, augment or diminish the output of nitrogen, sulphur, and phosphorus, and produce compounds which are difficult of elimination. By their immediate action they lower the resisting powers of our bodies and render us more susceptible to diseases and illnesses, and indirectly by modifying and altering the elimination of waste-products they are apt to engender a more or less marked feeling of lassitude, and in some cases they are accountable for inveterate headache.

In the case of our soldiers and sailors during an arduous and prolonged war it is quite possible that this indifference of ours to the use of preservatives in foods might be accountable for a series of reverses. During the South African war there was much invalidism amongst our troops, and although it is impossible to say to what extent this depended upon the presence of preservatives in the foods, yet, considering that one pound of preserved meat may not only be impregnated with sulphites, but may contain as much as 25 grains of boric acid, and that these preservatives exert a very prejudicial effect upon the metabolic processes in our bodies, it is highly probable that there would have been much less sickness if the use of preservatives in the rations had been prohibited.

Boric acid, which is to the consumer one of the most harmful of food preservatives, is very extensively used, and although on independent experimental evidence its use is prohibited in foods supplied for consumption both in Germany and the United States, yet, strange to say, the food advisers to our Local Government Board a few months ago recommended that as much as 19 grains in the pound weight of cream should be permitted.

